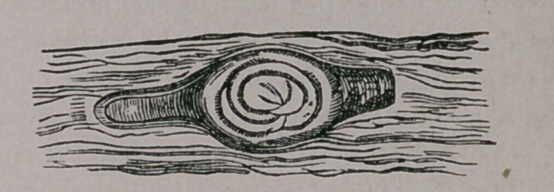# Trichina Spiralis

**Published:** 1876-10

**Authors:** 


### TRICHINA SPIRALIS.

The appearance of trichnia-disease during the month of May, last, in a neighboring county, created considerable excitement among the people of the locality where the disease prevailed, inasmuch as three persons died from the effects of the malady, and many more were rendered very ill. The disease originated among those who had eaten of ham procured at one place, further investigation showing that the meat had been obtained from one particular pig. Accordingly, the meat was subjected to microscopic test, when the peculiar spiral worm, known as *trichnia spiralis*, was at once recognized, the meat being thoroughly infected with them.

The discovery of the worms led to considerable discussion, those who were competent to judge claiming their presence in the meat, while others not skilled in the use of the microscope, declaring their nonexistence. To settle the matter, we were appealed to, as a microscopist, to decide whether the meat contained trichnia or no. The result of our investigation proved the meat to be thus affected, and the subsequent history of the symptoms of the persons who died, left no doubt in our mind of the cases having been true *trichiniasis*. So numerous were the inquiries made of us relative to the matter, that we concluded the subject to be of sufficient importance to warrant the preparation of this article for the BISTOURY, so that our readers who *will* persist in eating pork, may know the risk they run in so doing, as well as to enable them to inspect their meat and to recognize the mischievous little animal when they see him.

The perfectly developed entozoon is about one-eighteenth of an inch in length. The body is round, slightly bent upon itself, thicker behind than in front, head narrow, pointed, with a simple round opening for a mouth. They find their way into the intestinal canal of the hog, through the filthy food which he is in the habit of subsisting upon, and, after the second day of their introduction become full grown and ready to deposit their eggs, of which the female usually deposits from three to five hundred. Below we present a mature trichnia, magnified 300 diameters.

To the unaided eye he could not be distinguished, and the utmost care must be taken in preparing the specimen of meat to be examined, else will the microscope even, not reveal his presence to the unskilled eye.

In examining the suspected meat, be careful to cut your slice as thin as possible, remembering that the light must pass *through* the flesh in order to illuminate it properly. Spread the fragment upon your clean glass slide, and press it down with a thin glass film on top. Direct your reflected light from the little mirror beneath the stage of the microscope upon it, and then, with a power of fifty or seventy-five diameters, you will perceive, if the entozoon be present, a transparent, lemon-shaped cyst, in the center of which lies the trichnia, cuddled up as for a comfortable snooze. Remember that he is transparent, and of nearly the same color as the surrounding tissue. The accompanying cut will give you an idea as to his appearance in his cyst.

Take your specimen from the muscle and not the fat. They never inhabit the fat. Cut also in the direction of the muscular fibre, and you will procure much the best specimens.

After the eggs have been deposited by the mature female trichina, six days intervene before the young are hatched, when they immediately commence their migrations for the muscular tissues, passing through the walls of the intestines and abdominal cavity until they reach the muscles of their host. Here they curl themselves up in spiral form, the cysts form around them, and in fourteen days they have grown to their full size. The symptoms by which the presence of trichnia may be suspected are at first, before the parasites have laid their eggs, general discomfort, pain in stomach, and bowels, vomiting and diarrhœa. After the young are hatched, from the beginning of their wanderings to the muscles until they become permanently settled in their new home, the patient suffers from fever, great perspiration, swelling of the face, frequently extending to the entire body; inflamed eyes, intolerance of the light, pain over the eyes and pain also in moving the eyes, especially in looking upwards; diarrhœa and tenderness of the abdomen; severe pain in moving the muscles, especially of the neck and back, then of the arms and legs. If the chest muscles are invaded by the parasites, breathlessness and hiccough occur. Hoarseness and loss of voice, when they creep into the muscles of the larynx. In the last stage the sufferer is found upon his back with his legs drawn up, scarcely able to move or speak, while the fever increases, closely resembling typhoid fever; restlessness becomes more marked, delirium and then death comes to the relief of the sufferer. Dr. Zenker declares that the only important symptom of typhoid fever absent in trichnia disease, is the enlargement of the spleen, and believes that many of the so-called epidemics of typhoid fever might be directly traced to the results of eating trichinous pork.

Trichinæ have been found in other animals beside the hog. Virchow and Zenker have seen them in the flesh of cats, dogs, badgers, rats, hedgehogs, moles and swine. This is to be regretted, as those of swine eating proclivities will dislike to have their bill of fare so much curtailed.

In view of the great danger to which we are subjected in eating the flesh of the hog, can any reasonable excuse exist for our persisting in it, particularly when we have an abundance of other animals, cleanly in their nature and wholesome in flesh, from which to secure our food? We think not. But for those who *will* eat the miserable stuff, let us impress upon their minds the necessity of cooking it thoroughly, in order that they may deprive the trichnia of their wiggling capacity, before they enter the stomach.

### TO PREVENT DAMP FROM NORTH WALLS.

North walls are frequently damp from the absence of the drying effect of the sun; this defect may however be remedied by allowing ivy to grow over them. It acts both by preventing the access of rain, and by the rootlets absorbing moisture from the wall.

In very exposed situations the rain is frequently driven with such violence against the walls, as to penetrate through them, although the brick work is of considerable thickness. The evil may be obviated by dissolving three quarters of a pound of mottled soap in one gallon of boiling water, and spreading the hot solution steadily with a large flat brush, over the outer surface of the brickwork, taking care that it does not lather. This is to be allowed to dry for twenty-four hours, when a solution formed of a quarter of a pound of alum dissolved in two gallons of water, is to be applied in a similar manner over the coating of soap. The soap and alum mutually decompose each other, and form *an insoluble varnish* which the rain is unable to penetrate. The operation should be performed in dry settled weather.—*Mather's Circular.*

## Figures and Tables

**Figure f1:**
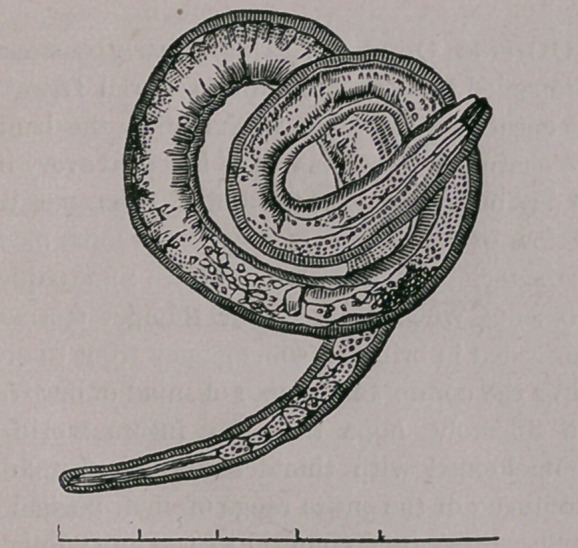


**Figure f2:**